# Rare FGFR fusion genes in cervical cancer and transcriptome‐based subgrouping of patients with a poor prognosis

**DOI:** 10.1002/cam4.6415

**Published:** 2023-08-03

**Authors:** Kengo Hiranuma, Yuka Asami, Mayumi Kobayashi Kato, Naoya Murakami, Yoko Shimada, Maiko Matsuda, Shu Yazaki, Erisa Fujii, Kazuki Sudo, Ikumi Kuno, Masaaki Komatsu, Ryuji Hamamoto, Hideki Makinoshima, Koji Matsumoto, Mitsuya Ishikawa, Takashi Kohno, Yasuhisa Terao, Atsuo Itakura, Hiroshi Yoshida, Kouya Shiraishi, Tomoyasu Kato

**Affiliations:** ^1^ Division of Genome Biology National Cancer Center Research Institute Tokyo Japan; ^2^ Department of Obstetrics and Gynecology Juntendo University Faculty of Medicine Tokyo Japan; ^3^ Department of Obstetrics and Gynecology Showa University School of Medicine Tokyo Japan; ^4^ Department of Gynecology National Cancer Center Hospital Tokyo Japan; ^5^ Department of Radiation Oncology National Cancer Center Hospital Tokyo Japan; ^6^ Department of Medical Oncology National Cancer Center Hospital Tokyo Japan; ^7^ Division of Medical AI Research and Development National Cancer Center Research Institute Tokyo Japan; ^8^ Cancer Translational Research Team RIKEN Center for Advanced Intelligence Project Tokyo Japan; ^9^ Tsuruoka Metabolomics Laboratory National Cancer Center Tsuruoka Japan; ^10^ Department of Diagnostic Pathology National Cancer Center Hospital Tokyo Japan; ^11^ Department of Clinical Genomics National Cancer Center Research Institute Tokyo Japan

**Keywords:** cervical cancer, fibroblast growth factor receptor 3, gene fusion, human papillomavirus, macrophage

## Abstract

**Background:**

Although cervical cancer is often characterized as preventable, its incidence continues to increase in low‐ and middle‐income countries, underscoring the need to develop novel therapeutics for this disease.This study assessed the distribution of fusion genes across cancer types and used an RNA‐based classification to divide cervical cancer patients with a poor prognosis into subgroups.

**Material and Methods:**

RNA sequencing of 116 patients with cervical cancer was conducted. Fusion genes were extracted using StarFusion program. To identify a high‐risk group for recurrence, 65 patients who received postoperative adjuvant therapy were subjected to non‐negative matrix factorization to identify differentially expressed genes between recurrent and nonrecurrent groups.

**Results:**

We identified three cases with FGFR3‐TACC3 and one with GOPC‐ROS1 fusion genes as potential targets. A search of publicly available data from cBioPortal (21,789 cases) and the Center for Cancer Genomics and Advanced Therapeutics (32,608 cases) showed that the FGFR3 fusion is present in 1.5% and 0.6% of patients with cervical cancer, respectively. The frequency of the FGFR3 fusion gene was higher in cervical cancer than in other cancers, regardless of ethnicity. Non‐negative matrix factorization identified that the patients were classified into four Basis groups. Pathway enrichment analysis identified more extracellular matrix kinetics dysregulation in Basis 3 and more immune system dysregulation in Basis 4 than in the good prognosis group. CIBERSORT analysis showed that the fraction of M1 macrophages was lower in the poor prognosis group than in the good prognosis group.

**Conclusions:**

The distribution of FGFR fusion genes in patients with cervical cancer was determined by RNA‐based analysis and used to classify patients into clinically relevant subgroups.

## Graphical abstract

Frequency of patients with FGFR3 fusion gene across cancer types consisting of our cohort and two public datasets (cBioPortal and C‐CAT).

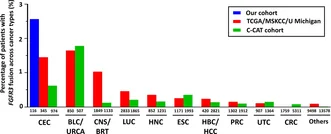

## 1 INTRODUCTION

Cervical cancer is the fourth most common cancer among women worldwide and the fourth most common cause of cancer‐related death.^1^ It is primarily caused by persistent infection by carcinogenic strains of the human papillomavirus (HPV), especially strains 16 and 18.^2^ Early‐stage cervical cancer refers to the International Federation of Gynecology and Obstetrics (FIGO) stages IA–IIA, which are usually treated by surgery alone. The recurrence rate of cervical cancer after surgical intervention ranges from 10% to 20%, with a typically unfavorable prognosis in the event of recurrence.^3^ The Gynecologic Oncology Group (GOG) defines tumor diameter (TD) >4 cm, lymphovascular space invasion (LVSI), and deep cervical stromal invasion (CSI) as intermediate‐risk pathological factors.^4^ The postoperative treatment of patients with risk factors includes radiotherapy or chemoradiotherapy^5^; however, the criteria for receiving postoperative chemoradiotherapy vary according to guidelines established in the United States,^6^ Europe,^7^ and Japan.^8^ Therefore, it is important to identify patients with a poor prognosis using different technologies such as genome analysis.

Comprehensive profiles of genomic alterations detected by whole‐exome and RNA‐sequencing in cervical cancer have been published by The Cancer Genome Atlas (TCGA).^9^ Mutations in *PIK3CA* are among the most frequently detected alterations in cervical cancer regardless of ethnicity.^10^, ^11^ Extensive whole‐exome sequencing of cervical carcinomas identified several important driver mutations and demonstrated that the development of cervical cancer is associated with specific somatic alterations in Caucasians.^9^, ^12^ These analyses also showed that HPV‐related cervical cancer is associated with structural aberrations and increased target gene expression. Despite the inclusion of Asian patients in previous studies, there are no reports using gene expression analysis to classify cervical cancer patients with a poor prognosis among the Asian population.^9^, ^12^ In addition, as the HPV types prevalent among Asian patients differ from those in other racial groups, it is important to analyze the Asian population separately.^13^ A recent study identified somatic mutations in DNA damage repair and chromatin remodeling pathway‐related genes as driver mutations.^14^ However, few reports have identified clinically relevant genetic alterations associated with therapeutic agents. By contrast, several pancancer studies have identified fusion genes as attractive therapeutic targets in many tumor types.^15^, ^16^ The *FGFR3* fusion gene has been detected in cervical cancer^17^; however, few studies have analyzed fusion genes using RNA‐sequencing.^18^

The prognostic stratification of patients with cervical cancer using RNA‐sequencing has not been widely attempted. Patients with positive pelvic nodes, positive surgical margin, and positive parametrium have a poor prognosis and require postoperative therapy.^19^ Although many biomarkers, such as *TP53* status and *STK11* mutations, correlate with a poor prognosis in cervical cancer,^10^, ^20^ a prognosis‐based classification for cervical cancer patients undergoing chemoradiotherapy remains to be established.

In this study, we performed RNA‐sequence analysis of 116 patients with cervical cancer to identify fusion genes as potential therapeutic targets. Public databases were used to evaluate the distribution of *FGFR* fusion genes across cancer types. Non‐negative matrix factorization (NMF) is used for clustering gene expression,^21^ identifying common mutation patterns,^12^ and classifying molecular subtypes.^22^ We used NMF clustering analysis to characterize the groups with poor prognostic factors in cervical cancer and to classify patients who received adjuvant radiotherapy or chemoradiotherapy based on genes with altered expression in the recurrence group.

## 2 METHODS

### 2.1 Patients and tumor samples

Frozen tumor tissues used in this study were obtained from 116 Japanese patients who were diagnosed with cervical cancer between 2002 and 2020 and underwent surgery as an initial treatment at the National Cancer Center Hospital, Tokyo, Japan. The clinical characteristics of the patients are summarized in Table 1. The tumors were restaged according to the 2018 International Federation of Obstetrics and Gynecology staging classification for cervical cancer because patients at high risk of recurrence (positive parametrium invasion or positive lymph node metastasis) undergo postoperative therapy in our hospital.^23^ The treatment strategy for each patient was discussed at the gynecological tumor board meeting of our hospital, which is attended by gynecologic oncologists, radiation oncologists, radiologists, pathologists, and medical oncologists. Pathologists carefully assessed all cases for the proportion of tumor cells and performed macro‐dissection when necessary. Abdominal radical hysterectomy was performed according to a previously reported method.^24^

**TABLE 1:** Characteristics in 116 patients with cervical cancer.

Variable	*n* (%)
Total	116
Age, median [range] (years)	43 [25–74]
Histological types	
Squamous cell carcinoma	66 (56.9)
Adenocarcinoma, HPV‐associated	24 (20.7)
Adenocarcinoma, Gastric‐type	7 (6.0)
Adenosquamous carcinoma	11 (9.5)
Neuroendocrine carcinoma	8 (6.9)
HPV genotype	
HPV 16	47 (40.5)
HPV 18	35 (30.2)
High‐risk HPV genotypes (31/35/39/45/52/56/58/59)	20 (17.3)
High‐risk HPV genotypes (ISH detected)	2 (1.7)
Negative	12 (10.3)
Pathological stage (FIGO 2018)	
IA1	1 (0.9)
IA2	2 (1.7)
IB1	2 (1.7)
IB2	27 (23.3)
IB3	21 (18.1)
IIA1	5 (4.3)
IIA2	2 (1.7)
IIB	7 (6.0)
IIIC1	43 (37.1)
IIIC2	6 (5.2)
Lymph node metastasis	
Positive	49
Negative	67
Parametrial involvement	
Positive	48
Negative	68
Maximum tumor diameter (cm)	
Median [range]	43 [14–100]
Adjuvant treatment	
RT alone	39
CCRT	19
Chemotherapy alone	7
Median follow‐up period [range] (months)	57.8 [0.9–134.6]

Abbreviations: CCRT, concurrent chemoradiation therapy; ISH, in situ hybridization; RT, radiation therapy.

### 2.2 DNA preparation and identification of somatic mutations by next‐generation sequencing

Genomic DNA was extracted from formalin‐fixed paraffin‐embedded (FFPE) or frozen tumor tissues using the QIAamp DNA FFPE tissue kit or the AllPrep DNA/RNA Mini Kit (Qiagen, Hilden, Germany) according to the manufacturer's instructions. Purified genomic DNA (50 ng) obtained from tumor tissues was used for library construction using the Ion AmpliSeqTM Cancer Hotspot Panel v2 (Thermo Fisher Scientific), which is designed to amplify 207 amplicons covering approximately 2800 catalogs of somatic mutations from 50 cancer‐related genes. Sequencing was performed using the Ion Proton platform (Thermo Fisher Scientific). For quality control, samples with a mean read depth of coverage >1000 and a base quality score of 20 (with ≤1% probability of being incorrect), which accounted for 90% of the total reads, were selected.

### 2.3 Total RNA extraction and RNA‐sequence analysis

RNA sequencing was performed using 1.1 μg of RNA isolated from snap‐frozen cancer tissues obtained from 116 uterine cervical cancer cases. Total RNA was purified from cancer tissues using the AllPrep DNA/RNA Mini Kit (Qiagen, Hilden, Germany). After quality assessment (RNA integrity number >6.0) using the Agilent 2100 Bioanalyzer (Agilent Technologies, Santa Clara, CA, USA), polyadenylated RNA libraries were generated using the TruSeq Stranded mRNA Library Prep Kit (Illumina, San Diego, CA, USA) and sequenced on the NovaSeq6000 platform using 2 × 150 bp paired‐end reads. Read mapping was performed using STAR version 2.4.2a^25^ with the human genome (GRCh38) and transcriptome data GENCODE version 31^26^ as reference datasets. STAR‐fusion was used to identify the actionable fusion genes according to the manual.^27^ Transcripts per million (TPM) values were calculated using the StringTie (2.0.4) program.^28^ The CIBERSORT program (https://cibersort.stanford.edu/)^29^ is a useful gene expression‐based arithmetic tool that uses a series of bar code genetic expression results (a “signature matrix” of 547 genes) to assess immunocyte constituents from bulk cancer specimens. For the precise quantification of the fraction of 22 immunocyte types in cervical cancer samples, standardized genetic expression measures such as TPM were sent to the CIBERSORT web portal, and the number of permutations was set to 1000.

### 2.4 RT‐PCR and Sanger sequencing

Total RNA (500 ng) was reverse‐transcribed to cDNA using Superscript III Reverse Transcriptase (Invitrogen). cDNA (corresponding to 10 ng total RNA) or 10 ng genomic DNA was subjected to PCR amplification using KAPA Taq DNA Polymerase (KAPA Biosystems). The reactions were carried out in a thermal cycler under the following conditions: 40 cycles of 95°C for 30 s, 60°C for 30 s, and 72°C for 2 min, with a final extension for 10 min at 72°C. The gene encoding GAPDH was used as a positive control for cDNA synthesis. The PCR products were directly sequenced in both directions using the BigDye Terminator kit and an Applied Biosystems DNA Sequencer (Applied Biosystems). The PCR primers used in the present study are listed in Table S1.

### 2.5 Distribution of *FGFR* fusion cases in multiple cancer types in different public databases

The frequency of *FGFR* fusions in diverse tumors was extracted from the cBioPortal for cancer genomics (http://cbioportal.org) using TCGA Pan‐cancer Atlas and the MSK‐IMPACT clinical sequencing cohort^30^ and metastatic solid cancer^31^ datasets. In total, 21,789 cancer cases were analyzed.

C‐CAT (https://www.ncc.go.jp/jp/c_cat/use/index.html) was established within the National Cancer Center in Japan to collect, analyze, and provide information on genomic medicine.^32^ Data of patients with *FGFR* fusion genes registered in the C‐CAT database (Ver.20220609) were collected; the dataset consisted of 32,608 Japanese pancancer patients. The Institutional Review Board of the National Cancer Center Research Institute approved this study (2020‐067). Detailed characteristics of 974 patients with cervical cancer in the C‐CAT cohort are shown in Table S2.

### 2.6 Identification of differentially expressed genes and molecular classification using NMF clustering analysis

The normalized count data were imported into Subio Platform v1.24.5853 (Subio Inc., Kagoshima, Japan: https://www.subioplatform.com.), and all subsequent analyses were executed using this software. To identify the poor prognosis group, 65 patients who received adjuvant therapy were selected to focus on patients with cervical cancer classified into the previously reported clinicopathological high‐risk group (lymph node metastasis/parametrial invasion).^8^, ^23^, ^33^, ^34^, ^35^ Ninety‐one differentially expressed genes (DEGs) were identified in 20 relapse cases compared with 45 nonrelapse cases; these genes showed >1.5‐fold differences in expression levels and had *p*‐values <0.1 (Figure 4A; Table S3).

NMF clustering analysis was used to identify the optimum number of clusters from cervical cancer RNA‐sequencing data. The R tool of NMF v‐0.23.0 was used for clustering using the 91 DEGs as input for clustering analysis. The number of clusters *k* was changed from 2 to 8, and the clustering process was repeated 500 times. The value of *k* that resulted in the second maximum cophenetic correlation coefficient was selected as the optimal number of clusters. Then, clustering was performed 500 times with the optimal *k* and random initialization to obtain the consensus matrix, samples, and associated genes. The clusters, which were divided into groups, were called the “Basis.”

### 2.7 Pathway enrichment analysis

Pathway enrichment analysis was performed using REACTOME.^36^ The 91 DEGs isolated from 65 cervical cancer cases that received postoperative adjuvant therapy were used (Table S3).

### 2.8 Data analysis using TCGA

RNA‐sequencing data and clinical data from 304 TCGA uterine cervical cancer samples were downloaded from the National Cancer Institute Genomic Data Commons (GDC) Data Portal (https://portal.gdc.cancer.gov/repository). STAR‐Fusion was applied to TCGA cervical cancer dataset to identify high‐confidence fusion transcripts.

### 2.9 Statistical analysis

Recurrence‐free survival (RFS), defined as the interval from the start of the first operation to any disease recurrence (local, regional, or distant), was calculated using the Kaplan–Meier method. Differences in outcomes were compared using the log‐rank test. Multivariate Cox proportional‐hazards models were used considering age, histology, tumor size, and adjuvant treatment. The data cut‐off date was November 17, 2021. Statistical analyses were performed using JMP version 15.0.0. (SAS Institute, NY, USA).

## 3 RESULTS

### 3.1 Patient characteristics

The patient characteristics are summarized in Table 1. The median age was 43 (range, 25–74) years. Of the 116 patients, 66 (56.9%) were diagnosed with squamous cell carcinoma (SCC), and 104 cervical tissue samples (89.7%) were positive for high‐risk HPV. The median follow‐up period was 57.8 (range, 0.9–134.6) months. Twenty‐seven patients experienced recurrence.

### 3.2 Detection of fusion genes in our cohort

**FIGURE 1:**
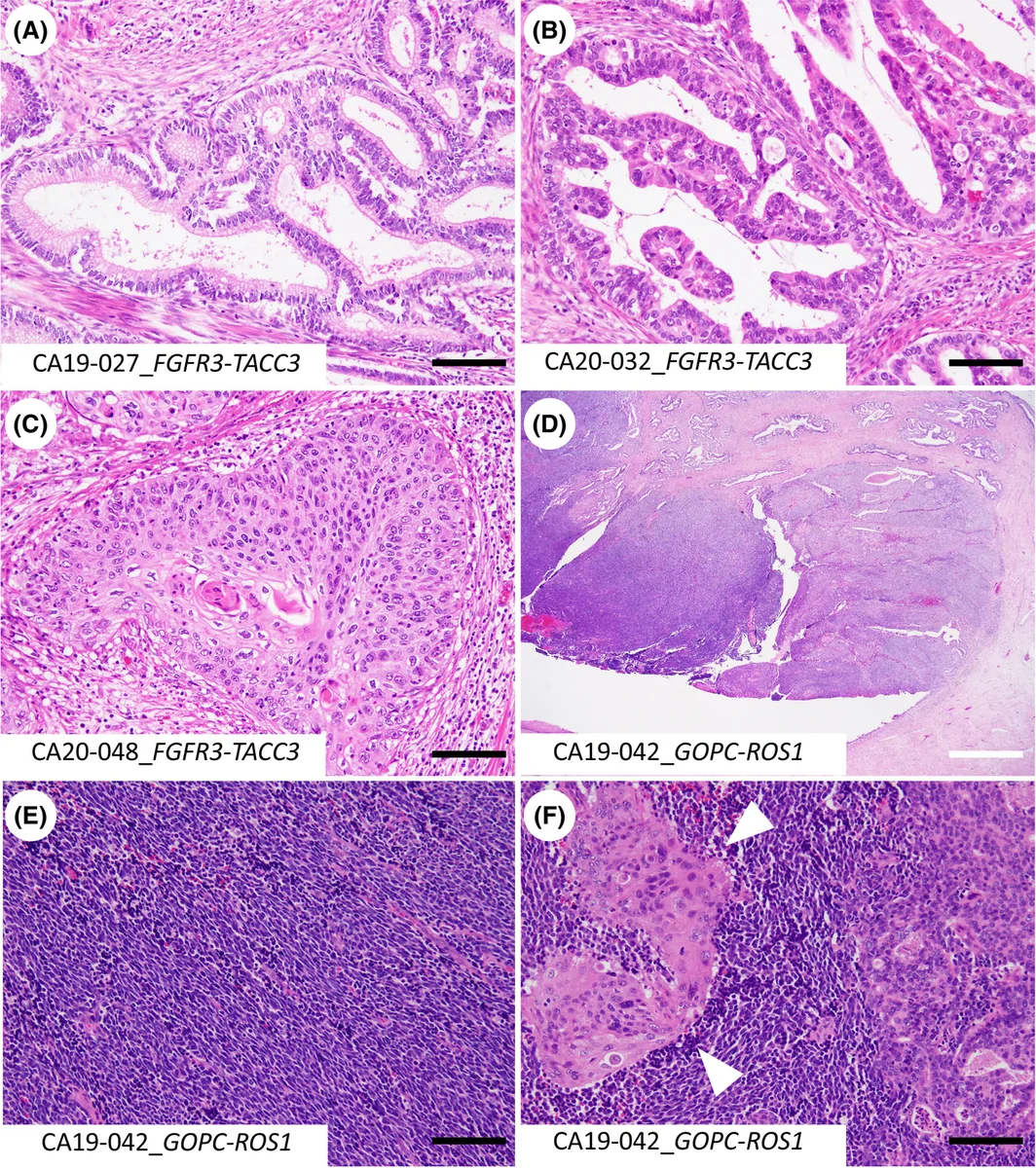
Histological findings of cervical cancers with *FGFR3‐TACC3* or *GOP3‐ROS1* fusion. Three cases of cervical cancer with *FGFR3‐TACC3* (A–C). Two cases show the typical morphology of HPV‐associated adenocarcinoma (A and B), whereas the other one is a case of HPV‐associated keratinizing squamous cell carcinoma (C). These cases show no morphological features distinct from those of cervical cancer without *FGFR3‐TACC3* fusion. The case with *GOP3‐ROS1* fusion is a case of small‐cell neuroendocrine carcinoma (SCNEC) with minor components of adenocarcinoma and squamous cell carcinoma (D–F). At lower magnification, the solid component of SCNEC (center) and an adenocarcinoma component (upper right) are observed (D). The SCNEC component is predominant in the tumor (E). There are small foci of squamous cell carcinoma (white arrowheads) and adenocarcinoma (right) with the background of the predominant SCNEC component (F). H&E staining (×200, A–C, E, and F; ×20, D). Black bars (A–C, E, F) and a white bar (D) indicate 100 and 500 μm, respectively.

**FIGURE 2:**
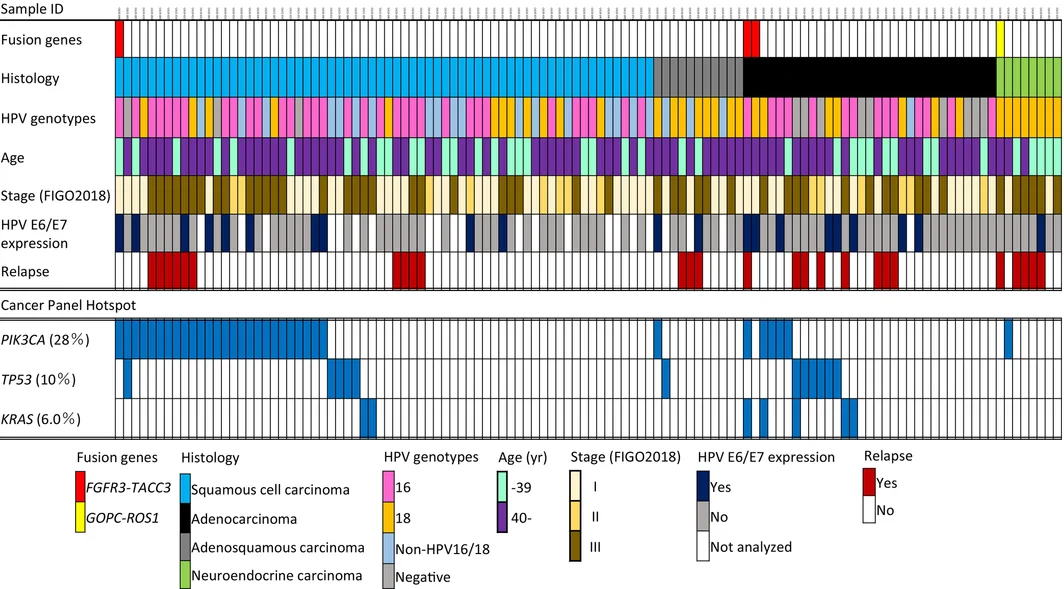
Clinicopathological features and mutation profiles of the present cohort (*n* = 116). Pathogenic/oncogenic mutations were detected in 56 cases (48%). The most frequent genomic alteration detected was *PIK3CA* (33 cases, 28%). Expression of E6/E7 in HPV 16/18 and mutation of *TP53* were almost mutually exclusive.

We identified three *FGFR3*‐*TACC3* fusion‐positive cases (2.6%), one *GOPC*‐*ROS1* fusion‐positive case (0.9%), and one *FGFR1‐ADAM9* fusion‐positive case (0.9%) using the STAR‐Fusion program^27^ in the Japanese cohort of 116 patients with cervical cancer (Table S4A). cDNA was synthesized using reverse‐transcription PCR; details are provided in the supporting materials and methods section (Figure S1A). The breakpoint in the fusion genes was identified by Sanger sequencing of cDNA (Figure S1B–E) synthesized using reverse‐transcription PCR; details are provided in the supporting materials and methods. In all *FGFR3*‐*TACC3* fusion‐positive and *GOPC*‐*ROS1* fusion‐positive cases, the kinase domain was preserved. In one *FGFR1*‐*ADAM9* fusion case, which was detected by STAR‐Fusion, the kinase domain was not preserved; thus, this fusion was assumed to be nonfunctional (Figure S1B). One *FGFR3*‐*TACC3* fusion‐positive case was histologically diagnosed as SCC, and two *FGFR3*‐*TACC3* fusion‐positive cases were histologically diagnosed as adenocarcinoma (Figure 1). No significant association between *FGFR3‐TACC3* fusions and clinicopathological features was observed (Figure 2). The *GOPC*‐*ROS1* fusion case was small‐cell neuroendocrine carcinoma. There was no association between genomic mutation frequencies and fusion genes, histology, and HPV genotypes; details are provided in the supporting materials and methods section (Figure 2).

### 3.3 Identification of somatic mutations by next‐generation sequencing

Targeted sequencing of the 116 cervical cancer specimens revealed pathogenic/oncogenic mutations in 42 cases (36%); details are provided in the Figure 2. *PIK3CA* was the most frequent genomic alteration detected in this study, with a frequency of 28% (Figure 2). Of the 27 cases with recurrence, 10 (37%) had at least one pathogenic/oncogenic mutation. The expression of HPV E6/E7 and mutation of *TP53* were almost mutually exclusive.

### 3.4 Frequency of pancancer with *FGFR* fusions registered in public databases

**FIGURE 3:**
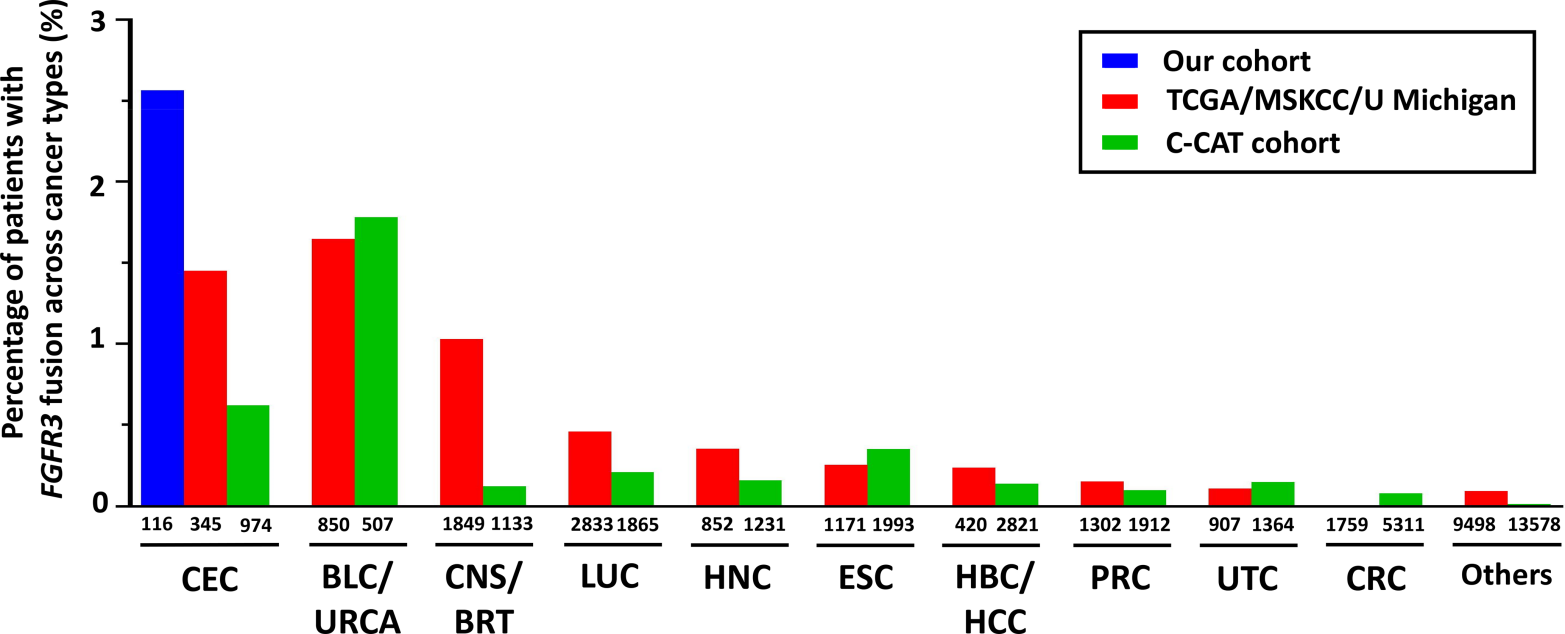
Frequency of patients with the *FGFR3* fusion gene in different cancer types in the present cohort and in two public datasets (cBioPortal and C‐CAT). CEC, cervical cancer; BLC, bladder cancer; URCA, urinary carcinoma; CNS, central nervous system; BRT, brain tumor; LUC, lung cancer; HNC, head and neck cancer; ESC, esophagogastric cancer; HBC, hepatobiliary cancer; BTC, biliary tract cancer; HCC, hepatocellular cancer; PRC, prostate cancer; UTC, uterine carcinoma; CRC, colorectal cancer.

We downloaded TCGA RNA‐sequencing data from 304 uterine cervical cancer samples from the National Cancer Institute GDC Data Portal. Of 304 TCGA cervical cancer samples, 5 *FGFR3*‐*TACC3* fusion‐positive samples were identified (Table S4B). The frequency of *FGFR3*‐*TACC3* fusion‐positive cervical cancer in the present cohort was similar to that of TCGA cohort (2.6% vs. 1.6%, respectively). We also evaluated the frequency of patients with *FGFR3* fusion‐positive cervical cancer in the C‐CAT database. Of 974 patients with advanced cervical cancer in the C‐CAT database, six *FGFR3* fusion‐positive cases were identified (Table S4C). The frequency of *FGFR3*‐*TACC3* fusion‐positive cervical cancer in the C‐CAT database was slightly lower (0.62%) than that in TCGA and the present cohorts (Figure 3).

Next, we investigated whether the frequency of *FGFR* fusion differs among different cancer types and ethnicities. First, we extracted the frequency of *FGFR* fusion in different tumors from 21,789 cases obtained from the cBioPortal for cancer genomics (http://cbioportal.org) consisting of TCGA Pan‐cancer Atlas studies, the MSK‐IMPACT clinical sequencing cohort,^30^ and metastatic solid cancer^31^ datasets (Table S5). *FGFR3* fusion cases were characteristic of bladder/urinary tract cancer and cervical cancer, with a high prevalence of 1.7% and 1.5%, respectively (Figure 3). Although *FGFR1* and *FGFR2* fusions were less frequent overall, *FGFR1* was relatively common in breast cancer (0.4%) and *FGFR2* in hepatobiliary/hepatocellular cancer (4.3%) (Figure S2A,S2B).

Next, we evaluated the frequency of *FGFR* fusion in the C‐CAT database consisting of 32,608 patients with advanced cancers (Table S6). *FGFR3* fusions were characteristic of bladder/urinary tract cancer (1.8%), central nervous system/brain tumors (1.2%), cervical cancer (0.6%), kidney cancer (0.4%), and esophagogastric/stomach cancer (0.4%). Five *FGFR3‐TACC3* fusion cases and one *FGFR3‐ADD1* fusion case were found in cervical cancer. *FGFR1* fusions were characteristic of head and neck cancer (0.3%). *FGFR2* fusions were characteristic of hepatobiliary/hepatocellular cancer (1.7%).

### 3.5 Molecular subtypes identified by NMF clustering analysis in our cohort

A TCGA study that performed hierarchical clustering in cervical cancer^9^ suggested the classification of cervical cancer cases into three groups: “keratin‐high,” characterized by high expression of *KRT13*, *SERPINB5*, *SERPINB13*, *TP63*, *KRT6A‐C*, *FAT2*, seven SPRRs, *ZNF750*, and *APOBEC3A*; “keratin‐low,” characterized by the expression of *TGFB1*, *TGFB2*, and *EPHB2*; and “adenocarcinoma,” characterized by the expression of *EPCAM*, *CLDN3*, *ERBB4*, *RAB17*, and *KRT18*. We performed hierarchical clustering based on the expression of 32 characteristic genes listed in Table S7 in the present cohort of 106 cervical cancer cases without neuroendocrine carcinoma. Three clusters (keratin‐high, keratin‐low, and adenocarcinoma) were created, as in the previous study (Figure S3). The percentage of the keratin‐low cluster was lower than that in the previous study, and this cluster could not be clearly classified into the three groups.

**FIGURE 4:**
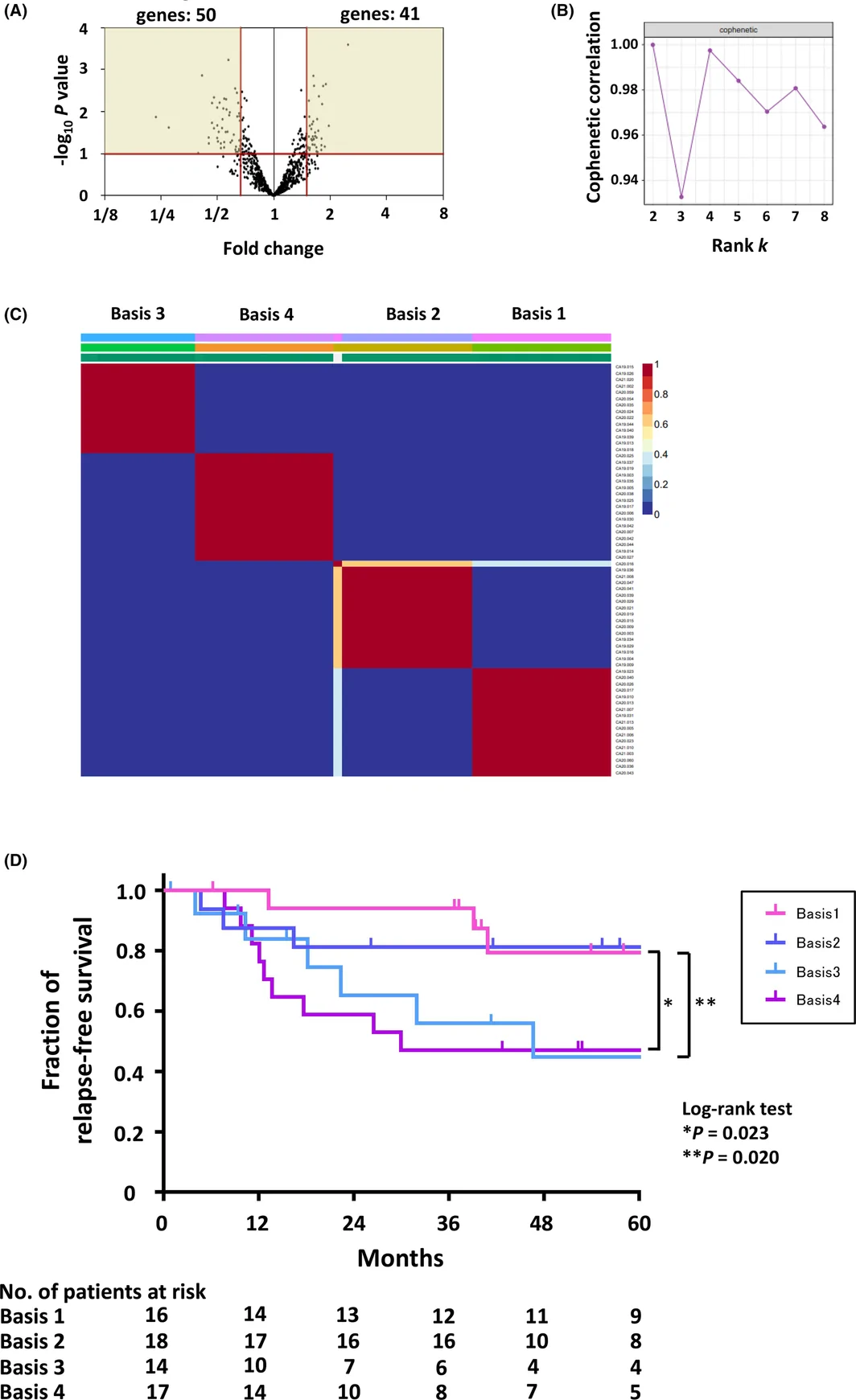
NMF clustering analysis of 65 cervical cancer cases. (A) There were 91 differentially expressed genes (DEGs) that differed by >1.5‐fold in their expression levels and had *p*‐values <0.1. Twenty relapse cases and forty‐five nonrelapse cases were analyzed. (B) Cophenetic correlation coefficients for the hierarchically clustered matrixes. High cophenetic correlations were observed for *k* = 2 and *k* = 4 classes. (C) The consensus matrix for *k* = 4 is shown. (D) Kaplan–Meier survival curves of the 65 patients with cervical cancer.

Identifying clinically relevant subtypes of cervical cancer based on molecular typing could facilitate the development of specific and efficient treatment options for patients with different subtypes. To identify the high‐risk group for recurrence in this cohort, we selected 65 patients with poor prognosis who received postoperative adjuvant therapy (Table S8). We identified 91 DEGs between relapse and nonrelapse cases (Figure 4A). Cophenetic correlation coefficients corresponding to the cervical cancer of consensus matrixes are shown in Figure 4B. Finally, based on the gene expression patterns in patients with cervical cancer, NMF clustering analysis was performed to distinctly classify all cervical cancer samples into four groups (Figure 4C). Bases 3/4 were more frequently relapsed clusters with non‐SCC tumors, and Bases 1/2 were less frequently relapsed clusters with SCC tumors (Figure S4). Kaplan–Meier survival curves were generated for patients with cervical cancer according to the Basis groups (Figure 4D). The 5 year RFS was significantly longer in the Basis 1 group than in the Basis 3 (*p* = 0.020) and Basis 4 (*p* = 0.023) groups. Multivariate analysis showed that Basis 3 tended to be associated with poor prognosis regarding RFS (hazard ratio = 2.0, *p* = 0.079) (Table S9).

### 3.6 Molecular subtypes identified by NMF clustering analysis in TCGA cohort

NMF clustering analysis in TCGA cohort was performed to validate the results. Cases with a follow‐up time of <1 year and stage I/IV were excluded to match TCGA cohort to the present cohort as closely as possible; finally, 89 cases from TCGA cohort were included in the analysis (Table S10). Cophenetic correlation coefficients corresponding to the cervical cancer of consensus matrixes are shown in Figure S5A. According to the gene expression patterns in patients with cervical cancer, NMF clustering analysis was performed to distinctly classify all cervical cancer samples into three groups (Figure S5B). Overall survival was slightly shorter in the Basis 1′ group than in the Basis 2′ and Basis 3′ groups (*p* = 0.2) (Figure S5C). Additionally, the Basis 1′ group formed a cluster with SCC and adenocarcinoma histology, and the Basis 2′/3′ groups formed clusters with SCC histology (Figure S6).

### 3.7 Characteristics of each Basis group according to pathway analysis

**FIGURE 5:**
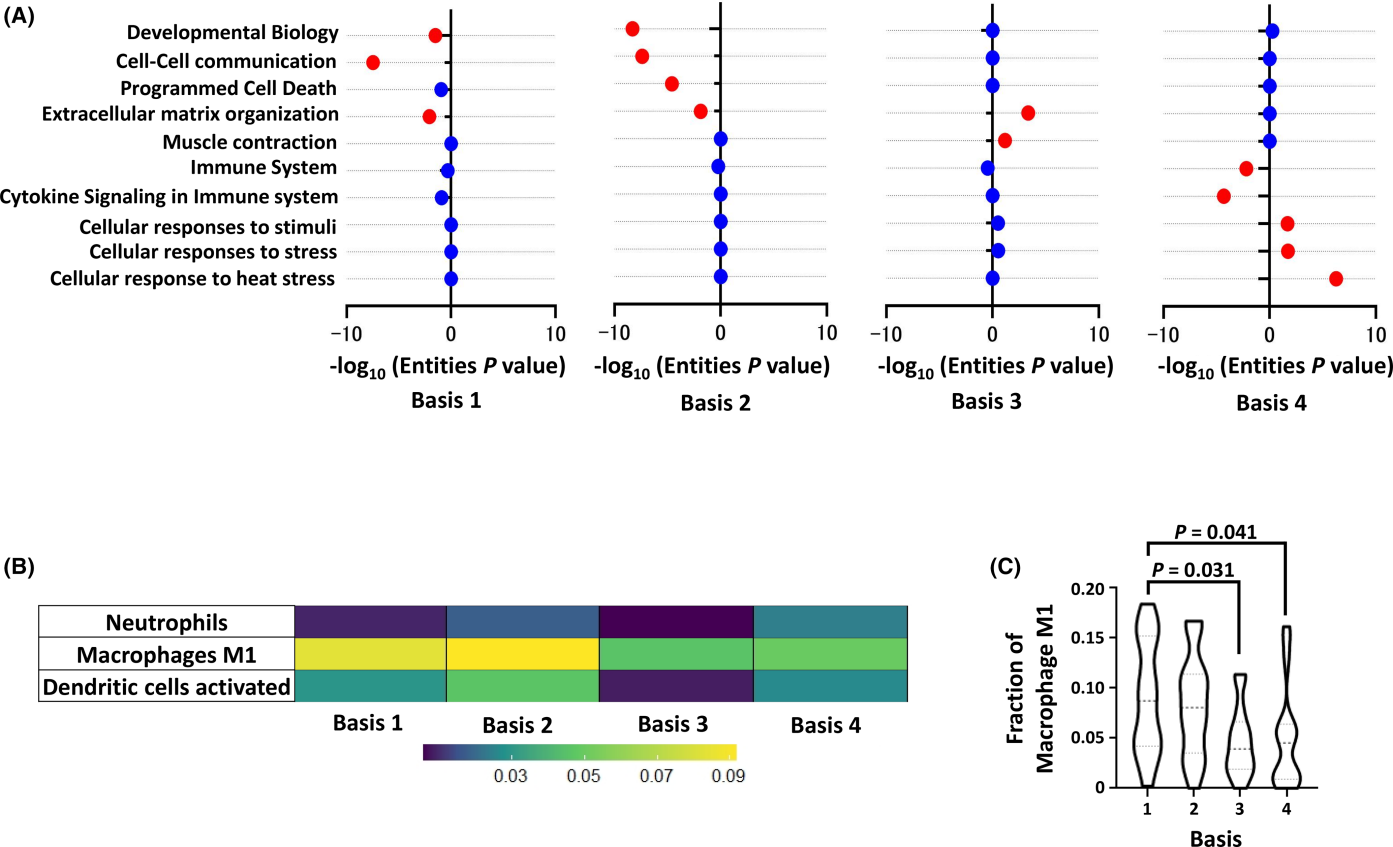
Immune cell infiltration characteristics in four Bases and results of pathway analysis. (A) REACTOME pathway enrichment analysis of each Basis. −log_10_ (entity *p*‐value) >1 or < −1 is indicated in red. (B) Heatmap of the immune cell fraction from the results of CIBERSORT analysis. Differences in immune cell fractions among Bases 1–4 were selected (one‐way ANOVA: *p* < 0.05). (C) Fraction of M1 macrophages in the four Bases.

Pathway enrichment analysis was performed using REACTOME^36^ to extract features of each Basis group in the cohort determined by NMF clustering. The gene sets in each Basis group are shown in Table S11, and the results of pathway enrichment analysis are shown in Figure 5A. In the Basis 1 group, downregulated genes were related to cell junction organization and cell–cell communication pathways (Table S12). In the Basis 2 group, downregulated genes were related to keratinization and apoptotic cleavage of cell adhesion proteins (Table S13). In the Basis 3 group, upregulated genes were related to posttranslational protein phosphorylation and *RUNX3*‐regulated *YAP1*‐mediated transcription pathways (Table S14A), whereas downregulated genes were related to extracellular matrix (ECM) proteoglycans and nonintegrin membrane‐ECM interactions (Table S14B). In the Basis 4 group, upregulated genes were related to heat shock factor 1 (HSF1)‐dependent transactivation and HSF1 activation pathways (Table S15A), whereas downregulated genes were related to interferon‐alpha/beta signaling, interferon signaling, and interferon‐gamma signaling pathways (Table S15B). Collectively, the Basis 1 and 2 groups showed downregulation of cell–cell communication‐related pathways and a favorable prognosis. By contrast, Basis 3 showed dysregulation of ECM dynamics related to cancer prognosis, and Basis 4 had dysregulation of immune system‐related pathways, such as antiviral responses, and were related to an unfavorable prognosis.

Next, we conducted pathway analysis for each Basis group in 89 cases from TCGA cohort. Immune‐related pathways were downregulated in the poor prognosis group, basis 1′ (Table S16). The downregulation of interferon‐alpha/beta signaling, interferon signaling, and interferon‐gamma signaling pathways, such as antiviral responses, was observed in both TCGA and the present cohort in association with a poor prognosis.

### 3.8 Differences among the four bases and immune cell fractions based on CIBERSORT analysis

To determine whether differences in antiviral activity differentially affect immune cell fractions, we performed immune cell fraction analysis using CIBERSORT.^29^ The average of immune cell fractions in each Basis group was used to generate a heatmap (Figure 5B). M1 macrophages exhibited a significantly lower expression in both Bases 3/4 and Basis 1′, which are associated with a poor prognosis (Figure 5C; Figure S5D).

## 4 DISCUSSION

In this study, we performed RNA sequencing of 116 cervical cancer samples and detected four cases with fusion genes involving *FGFR3* or *ROS1* as potential therapeutic targets. *FGFR3‐TACC3* fusions and *GOPC‐ROS1* fusions are commonly observed among exon‐exon fusions in other cancers.^37^, ^38^ To the best of our knowledge, this is the first report to identify *GOPC*‐*ROS1* fusion in cervical cancer. *FGFR3* fusion was detected in cervical cancer regardless of ethnicity and histology. Although most counterparts of *FGFR3* were *TACC3*, one case of *ADD1* was detected as a novel counterpart from the C‐CAT data. The distribution of *FGFR3* fusion differed from that of *FGFR1* and *FGFR2* fusions, indicating that the distribution of *FGFR* fusion genes is cancer type‐dependent. To identify the groups with a poor prognosis, potential prognostic factors in patients who underwent adjuvant therapy were detected by RNA‐seq.

*FGFRs* are a family of four receptor tyrosine kinases: *FGFR1*–*FGFR4*.^39^ Although these receptors are encoded by different genes, they share a highly conserved DNA sequence.^40^ The diversity among *FGFRs* is attributed to the alternative splicing of the mRNA sequence that produces the extramembranous domain.^41^ These differences may also be related to differences in the site of cancer onset. *FGFR3*‐*TACC3* is formed by an intrachromosomal translocation of chromosome 4 between *FGFR3* and *TACC3*. FGFRs are normally activated by the binding of FGF‐heparin to monomeric FGFRs, which leads to dimerization and transphosphorylation of the cytoplasmic tyrosine kinase domain. This activates several downstream signaling pathways, including the MAPK pathway.^42^ Aberrant activation of FGFR signaling has been implicated in cell proliferation and tumorigenesis. TACC3 has a coiled‐coil domain at its C terminus that results in TACC3 dimerization and even multimerization under certain circumstances.^43^, ^44^, ^45^ The TACC domain is also present in FGFR3‐TACC3 fusions and is thought to constitutively activate FGFR3 signaling, as well as other fusion mechanisms involving receptor tyrosine kinases.^46^, ^47^ FGFR3‐TACC3 fusion proteins are oncogenic factors that can be targeted by small molecule kinase inhibitors.^42^ Multiple clinical trials are ongoing to evaluate the role of *FGFR*‐targeted therapy in patients with advanced solid tumors with *FGF/FGFR* alterations. The response rate is higher in patients with *FGFR* fusions/rearrangements/mutations than in those without *FGFR* alterations.^48^ Patients with cervical SCC harboring *FGFR3‐TACC3* fusion show a partial response to AZD5457 (*FGFR1‐3* inhibitor).^49^ Similar therapeutic targeting agents could be effective in patients with cervical cancer with *FGFR* fusion genes. The frequency of *FGFR3* fusion in the present cohort was 2.6% compared with 1.4% in the data extracted from cBioPortal. However, the frequency of *FGFR3* fusion in the C‐CAT database, which consists of only advanced cervical cancer cases, is 0.62%. Considering that the frequency of *FGFR3* fusion is lower in advanced cases, the prognosis of patients with cervical cancer with this fusion may be better.

We also identified the *GOPC‐ROS1* fusion, which is the first case detected in cervical cancer. *ROS1* fusion genes have been detected in other cancer types, such as glioma and lung adenocarcinoma.^50^, ^51^ ROS1 inhibition is an effective strategy for the treatment of patients with *ROS1*‐rearranged nonsmall‐cell lung cancer (NSCLC). Crizotinib, a ROS1 tyrosine kinase inhibitor, has shown promising results in patients with NSCLC harboring *ROS1* rearrangement.^52^, ^53^ Moreover, the case with *GOPC‐ROS1* fusion in this study had neuroendocrine carcinoma, a highly aggressive tumor with a limited number of therapeutic targets.^54^

NMF clustering analysis was performed using RNA‐Seq to identify poor prognosis groups, and cervical cancer patients who received adjuvant therapy were classified into four groups. Bases 1/2, which mainly included SCC cases, showed a longer RFS, whereas Bases 3/4, which mainly included non‐SCC cases, had a poor prognosis. Pathway enrichment analysis using the gene set that was used for classification showed that ECM dynamics was dysregulated in Base 3, which is classified as a poor prognosis group, and immune system‐related pathways were dysregulated in Base 4. Although the composition and organization of the ECM are spatiotemporally regulated to control cell behavior and differentiation, dysregulation of ECM dynamics leads to the development of diseases such as cancer; the chemical cues presented by the ECM are considered key drivers of cancer development and progression.^55^ Thus, the dysregulation of ECM dynamics in Base 3 may be associated with a poor prognosis. On the other hand, the macrophage fraction was reduced in Bases 3/4. Macrophages are phenotypic heterogenic cells. Classically activated macrophages (M1), which are polarized by lipopolysaccharide (LPS) either alone or in combination with interferon (IFN)‐γ, produce proinflammatory cytokines and have antitumor effects.^56^, ^57^ In Basis 4, the decrease in the M1 macrophage fraction and the downregulation of the interferon signaling pathway suggest that the antitumor capacity was decreased. This is a good prognostic factor in esophageal SCC.^58^

We performed NMF clustering analysis using TCGA dataset. However, the same NMF clustering could not be performed in the present cohort because it differed significantly from TCGA dataset. These differences include the lack of recurrence data and information on adjuvant treatment, distribution of histological type, and race of the patients. However, the downregulation of immune‐related pathways in the poor prognosis group in both TCGA and the present cohorts may have influenced the elimination of cervical cancer with HPV infection. On the other hand, there are reports using RNA‐Seq to classify TCGA cohort into three groups. Because Basis 1–4 and the gene sets used to classify the three groups had only four genes in common, namely, *TP63*, *KRT6A*, *SERPINB5*, and *SERPINB13*, no association was found between Basis 1–4 and the three groups.

The present study had several limitations. First, because there are few studies of Asian and Caucasian cervical cancer that include detailed clinical information, such as postoperative chemotherapy and RFS using RNA‐sequencing, it is difficult to perform accurate validation studies for the NMF clustering analysis. Second, because of the small number of cases in this study, we were unable to adequately adjust for confounding factors such as histology and treatment history, and further validation is needed.

In conclusion, RNA sequence‐based analysis identified and confirmed the presence of *FGFR3*‐*TACC3* fusion genes in cervical cancer and other cancer types. NMF clustering analysis revealed different mRNA expression patterns in cervical cancer and identified a subgroup with a poor prognosis.

## AUTHOR CONTRIBUTIONS

**Kengo Hiranuma:** Data curation (equal); formal analysis (equal); investigation (equal); methodology (equal); project administration (equal); visualization (equal); writing – original draft (equal). **Yuka Asami:** Data curation (equal); writing – review and editing (equal). **Mayumi Kobayashi Kato:** Writing – review and editing (equal). **Naoya Murakami:** Writing – review and editing (equal). **Yoko Shimada:** Data curation (equal); resources (equal). **Maiko Matsuda:** Data curation (equal). **Shu Yazaki:** Writing – review and editing (equal). **Erisa Fujii:** Writing – review and editing (equal). **Kazuki Sudo:** Supervision (equal). **Ikumi Kuno:** Supervision (equal). **Masaaki Komatsu:** Supervision (equal). **Ryuji Hamamoto:** Supervision (equal). **Hideki Makinoshima:** Supervision (equal). **Koji Matsumoto:** Supervision (equal). **Mitsuya Ishikawa:** Supervision (equal). **Takashi Kohno:** Project administration (equal); supervision (equal). **Yasuhisa Terao:** Supervision (equal). **Atsuo Itakura:** Supervision (equal). **Hiroshi Yoshida:** Supervision (equal); writing – review and editing (equal). **Kouya Shiraishi:** Conceptualization (equal); funding acquisition (equal); methodology (equal); resources (equal); software (equal); supervision (equal); writing – review and editing (equal). **Tomoyasu Kato:** Supervision (equal).

## FUNDING INFORMATION

This work was supported by a Grant‐in‐Aid for Young Scientists (B) Number 20K18207 and 19K16572, a Grant‐in‐Aid for Scientific Research (C) Number 20K09636, and the National Cancer Center Research and Development Fund (2020‐J‐2, NCC Biobank, and NCC Core Facility).

## CONFLICT OF INTEREST STATEMENT

All other authors have no conflicts of interest to declare.

## ETHICS STATEMENT

The National Cancer Center Research Institute's Institutional Review Board approved this study (2017‐136).

## PATIENT CONSENT

All patients provided written informed consent.

## CLINICAL TRIAL REGISTRATION

Not applicable

## ANIMAL STUDIES

Not applicable

## Supporting information


Figure S1.
Figure S2.
Figure S3.
Figure S4.
Figure S5.
Figure S6.

Table S1.
Table S2.
Table S3.
Table S4.
Table S5.
Table S6.
Table S7.
Table S8.
Table S9.
Table S10.
Table S11.
Table S12.
Table S13.
Table S14.
Table S15.
Table S16.

**Data S1.** Supporting material and methods

## Data Availability

The data that support the findings of this study and further information are available from the corresponding author upon reasonable request.
