# Supplementary material for: Rare FGFR fusion genes in cervical cancer and transcriptome‐based subgrouping of patients with a poor prognosis
Source: Cancer Med. 2023 Aug 3;12(17):17835–48. doi: 10.1002/cam4.6415 (PMC10524028; doi:10.1002/cam4.6415)
Supplement: Supplementary file 1 — Figure S1. Figure S2. Figure S3. Figure S4. Figure S5. Figure S6. [file CAM4-12-17835-s001.pptx]

## Slide 1
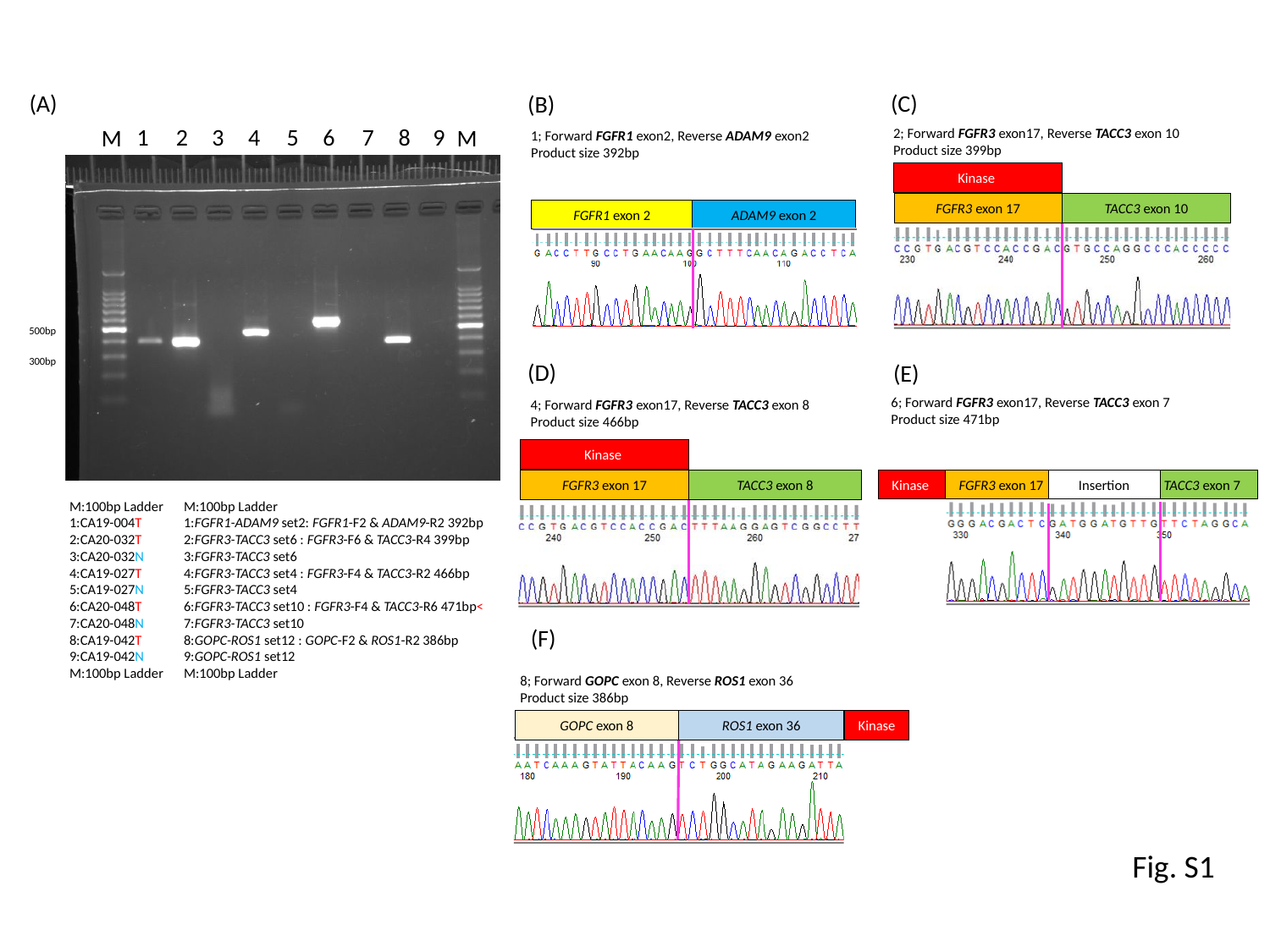

(A)
(C)
(B)
1
2
3
4
5
6
7
8
9
M
M
2; Forward FGFR3 exon17, Reverse TACC3 exon 10
Product size 399bp
Kinase
FGFR3 exon 17
TACC3 exon 10
1; Forward FGFR1 exon2, Reverse ADAM9 exon2
Product size 392bp
FGFR1 exon 2
ADAM9 exon 2
500bp
300bp
(D)
(E)
6; Forward FGFR3 exon17, Reverse TACC3 exon 7
Product size 471bp
Kinase
FGFR3 exon 17
Insertion
TACC3 exon 7
4; Forward FGFR3 exon17, Reverse TACC3 exon 8
Product size 466bp
Kinase
FGFR3 exon 17
TACC3 exon 8
M:100bp Ladder
1:CA19-004T
2:CA20-032T
3:CA20-032N
4:CA19-027T
5:CA19-027N
6:CA20-048T
7:CA20-048N
8:CA19-042T
9:CA19-042N
M:100bp Ladder
M:100bp Ladder
1:FGFR1-ADAM9 set2: FGFR1-F2 & ADAM9-R2 392bp
2:FGFR3-TACC3 set6 : FGFR3-F6 & TACC3-R4 399bp
3:FGFR3-TACC3 set6
4:FGFR3-TACC3 set4 : FGFR3-F4 & TACC3-R2 466bp
5:FGFR3-TACC3 set4
6:FGFR3-TACC3 set10 : FGFR3-F4 & TACC3-R6 471bp<
7:FGFR3-TACC3 set10
8:GOPC-ROS1 set12 : GOPC-F2 & ROS1-R2 386bp
9:GOPC-ROS1 set12
M:100bp Ladder
(F)
8; Forward GOPC exon 8, Reverse ROS1 exon 36
Product size 386bp
GOPC exon 8
ROS1 exon 36
Kinase
Fig. S1

## Slide 2
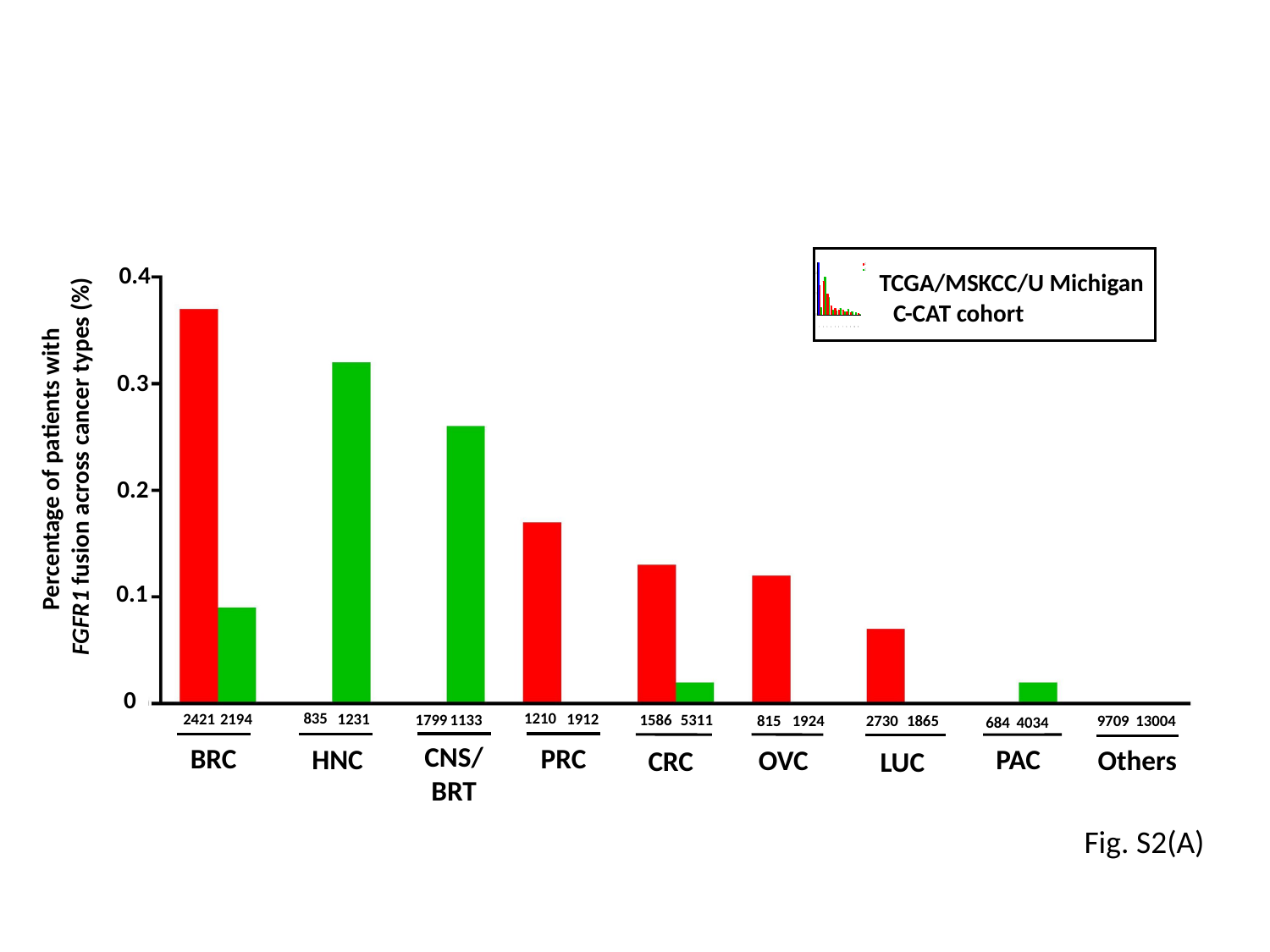

TCGA/MSKCC/U Michigan
C-CAT cohort
0.4
0.3
Percentage of patients with
FGFR1 fusion across cancer types (%)
0.2
0.1
0
1210
835
2421
2194
1231
1912
5311
1586
1799
1133
815
9709
13004
1865
2730
1924
4034
684
CNS/BRT
PRC
BRC
HNC
PAC
OVC
Others
CRC
LUC
Fig. S2(A)

## Slide 3
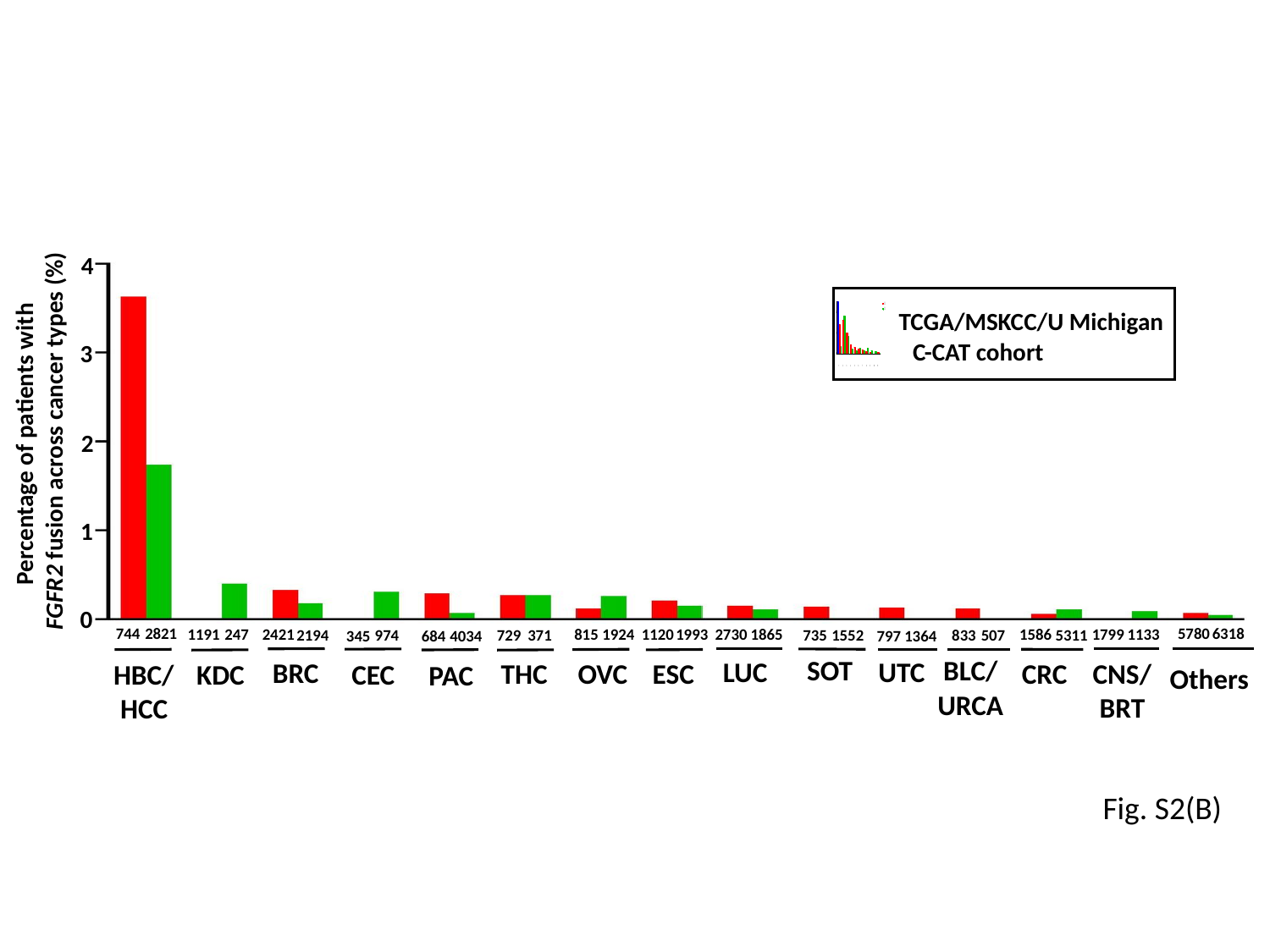

4
TCGA/MSKCC/U Michigan
C-CAT cohort
3
Percentage of patients with
FGFR2 fusion across cancer types (%)
2
1
0
2821
5780
6318
744
1120
1191
1993
2730
1865
1799
1133
2421
1924
1586
815
247
371
5311
2194
735
833
729
1552
507
974
345
684
797
1364
4034
SOT
BLC/URCA
LUC
UTC
BRC
THC
OVC
ESC
CRC
CNS/BRT
KDC
HBC/
HCC
CEC
PAC
Others
Fig. S2(B)

## Slide 4
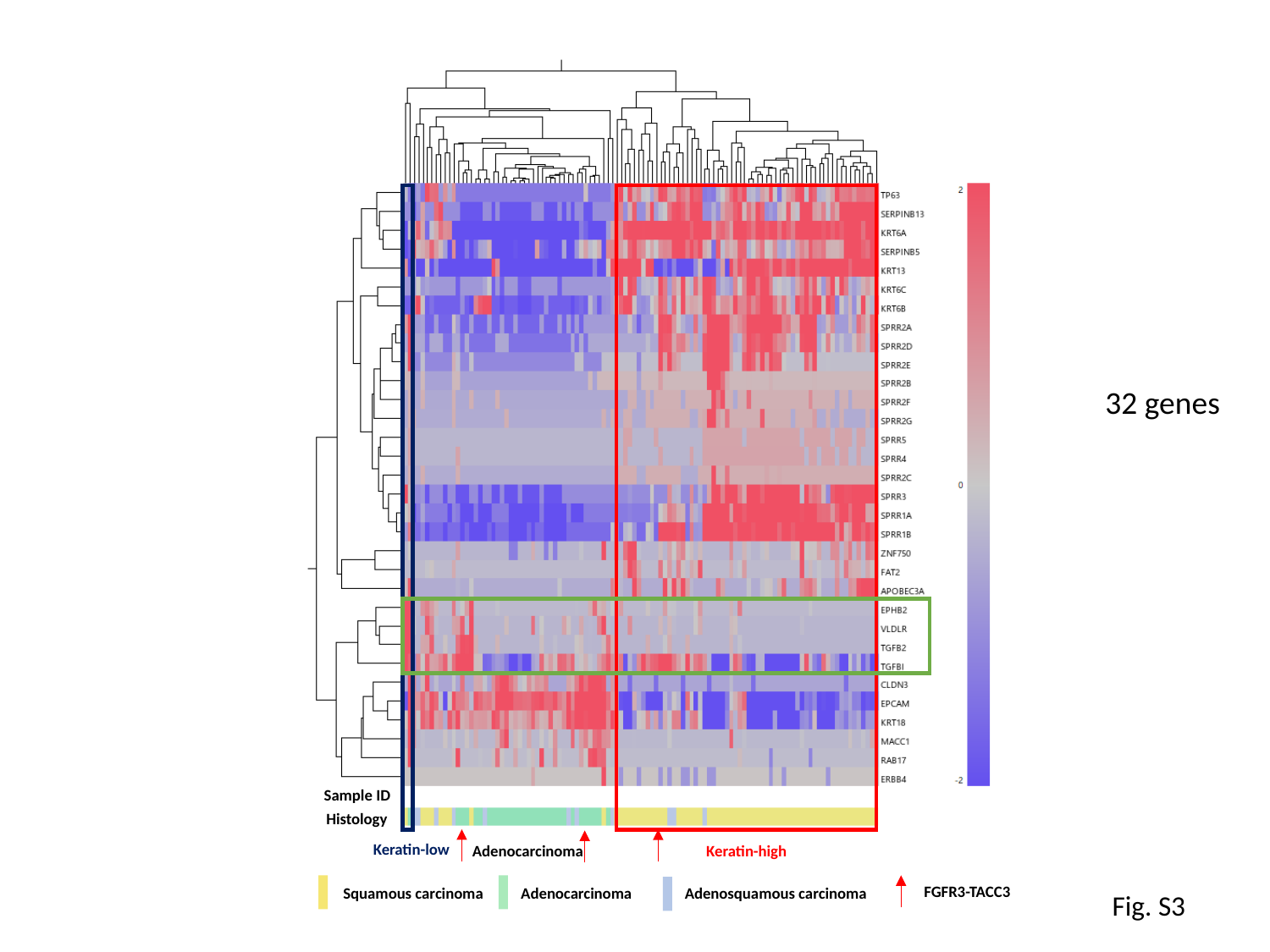

Sample ID
Histology
Keratin-low
Adenocarcinoma
Keratin-high
32 genes
FGFR3-TACC3
Adenosquamous carcinoma
Squamous carcinoma
Adenocarcinoma
Fig. S3

## Slide 5
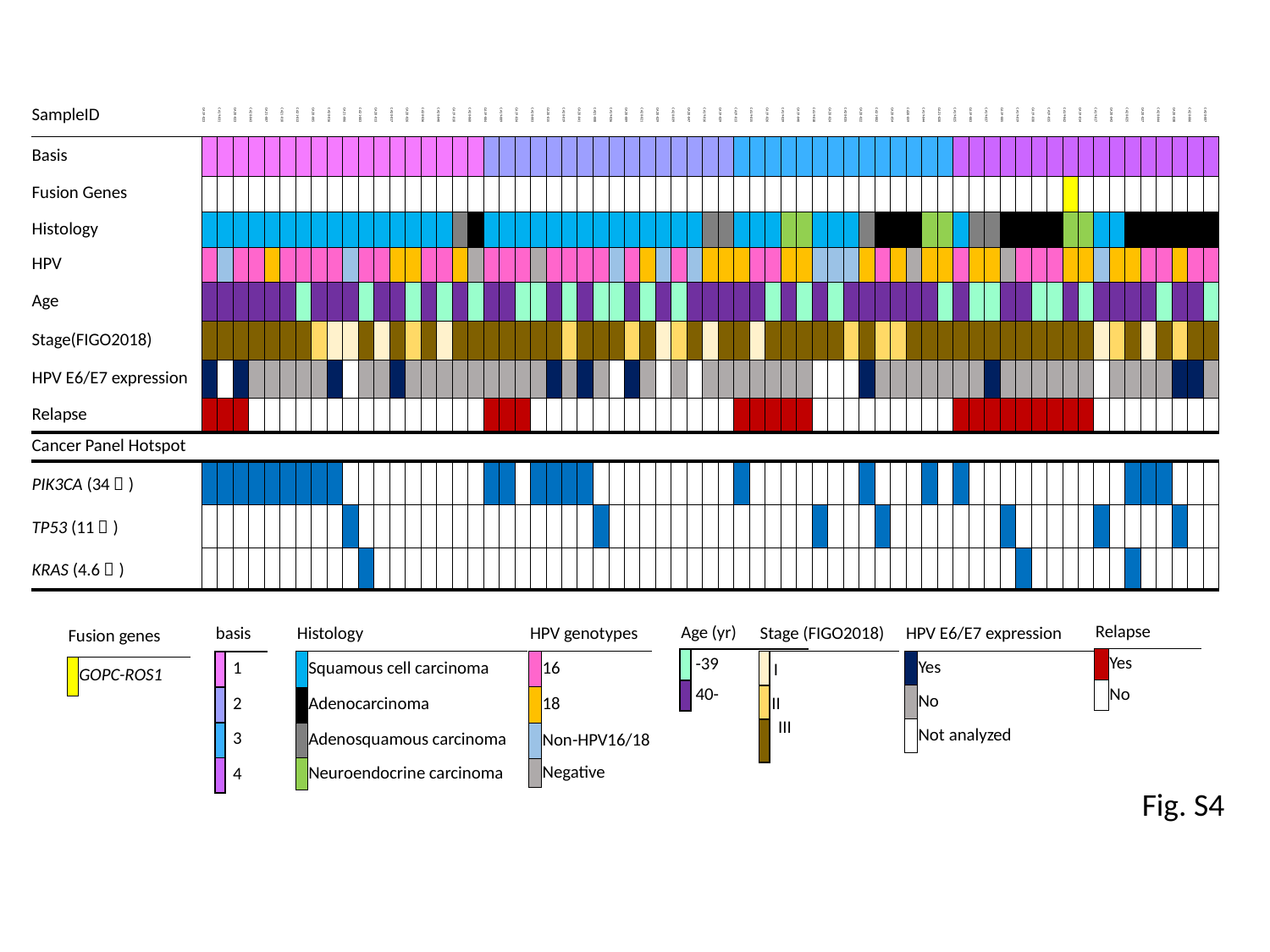

| SampleID | CA19-023 | CA19-031 | CA20-023 | CA20-043 | CA21-007 | CA21-010 | CA21-013 | CA20-005 | CA20-016 | CA21-006 | CA21-003 | CA20-013 | CA20-017 | CA20-026 | CA20-036 | CA20-040 | CA19-010 | CA20-060 | CA19-004 | CA19-009 | CA19-034 | CA20-003 | CA20-015 | CA20-019 | CA20-041 | CA21-008 | CA19-036 | CA20-009 | CA20-021 | CA20-029 | CA20-039 | CA20-047 | CA19-016 | CA19-029 | CA19-013 | CA19-015 | CA19-026 | CA19-039 | CA19-040 | CA19-018 | CA20-024 | CA20-035 | CA20-022 | CA21-002 | CA20-054 | CA20-059 | CA19-044 | CA21-020 | CA19-025 | CA19-003 | CA19-037 | CA19-005 | CA19-019 | CA19-030 | CA19-035 | CA19-042 | CA19-014 | CA19-017 | CA20-042 | CA20-025 | CA20-027 | CA20-044 | CA20-038 | CA20-006 | CA20-007 |
| --- | --- | --- | --- | --- | --- | --- | --- | --- | --- | --- | --- | --- | --- | --- | --- | --- | --- | --- | --- | --- | --- | --- | --- | --- | --- | --- | --- | --- | --- | --- | --- | --- | --- | --- | --- | --- | --- | --- | --- | --- | --- | --- | --- | --- | --- | --- | --- | --- | --- | --- | --- | --- | --- | --- | --- | --- | --- | --- | --- | --- | --- | --- | --- | --- | --- |
| Basis | | | | | | | | | | | | | | | | | | | | | | | | | | | | | | | | | | | | | | | | | | | | | | | | | | | | | | | | | | | | | | | | | |
| Fusion Genes | | | | | | | | | | | | | | | | | | | | | | | | | | | | | | | | | | | | | | | | | | | | | | | | | | | | | | | | | | | | | | | | | |
| Histology | | | | | | | | | | | | | | | | | | | | | | | | | | | | | | | | | | | | | | | | | | | | | | | | | | | | | | | | | | | | | | | | | |
| HPV | | | | | | | | | | | | | | | | | | | | | | | | | | | | | | | | | | | | | | | | | | | | | | | | | | | | | | | | | | | | | | | | | |
| Age | | | | | | | | | | | | | | | | | | | | | | | | | | | | | | | | | | | | | | | | | | | | | | | | | | | | | | | | | | | | | | | | | |
| Stage(FIGO2018) | | | | | | | | | | | | | | | | | | | | | | | | | | | | | | | | | | | | | | | | | | | | | | | | | | | | | | | | | | | | | | | | | |
| HPV E6/E7 expression | | | | | | | | | | | | | | | | | | | | | | | | | | | | | | | | | | | | | | | | | | | | | | | | | | | | | | | | | | | | | | | | | |
| Relapse | | | | | | | | | | | | | | | | | | | | | | | | | | | | | | | | | | | | | | | | | | | | | | | | | | | | | | | | | | | | | | | | | |
| Cancer Panel Hotspot | | | | | | | | | | | | | | | | | | | | | | | | | | | | | | | | | | | | | | | | | | | | | | | | | | | | | | | | | | | | | | | | | |
| PIK3CA (34％) | | | | | | | | | | | | | | | | | | | | | | | | | | | | | | | | | | | | | | | | | | | | | | | | | | | | | | | | | | | | | | | | | |
| TP53 (11％) | | | | | | | | | | | | | | | | | | | | | | | | | | | | | | | | | | | | | | | | | | | | | | | | | | | | | | | | | | | | | | | | | |
| KRAS (4.6％) | | | | | | | | | | | | | | | | | | | | | | | | | | | | | | | | | | | | | | | | | | | | | | | | | | | | | | | | | | | | | | | | | |
| Fusion genes | | | |
| --- | --- | --- | --- |
| | GOPC-ROS1 | | |
| HPV E6/E7 expression | | | | | |
| --- | --- | --- | --- | --- | --- |
| | Yes | | | | |
| | No | | | | |
| | Not analyzed | | | | |
| basis | | |
| --- | --- | --- |
| | 1 | |
| | 2 | |
| | 3 | |
| | 4 | |
| Histology | | | | | | | | | | |
| --- | --- | --- | --- | --- | --- | --- | --- | --- | --- | --- |
| | Squamous cell carcinoma | | | | | | | | | |
| | Adenocarcinoma | | | | | | | | | |
| | Adenosquamous carcinoma | | | | | | | | | |
| | Neuroendocrine carcinoma | | | | | | | | | |
| Relapse | | | |
| --- | --- | --- | --- |
| | Yes | | |
| | No | | |
| HPV genotypes | | | | | |
| --- | --- | --- | --- | --- | --- |
| | 16 | | | | |
| | 18 | | | | |
| | Non-HPV16/18 | | | | |
| | Negative | | | | |
| Age (yr) | | | | | | |
| --- | --- | --- | --- | --- | --- | --- |
| | -39 | | | | | |
| | 40- | | | | | |
| Stage (FIGO2018) | | |
| --- | --- | --- |
| | I | |
| | II | |
| | III | |
Fig. S4

## Slide 6
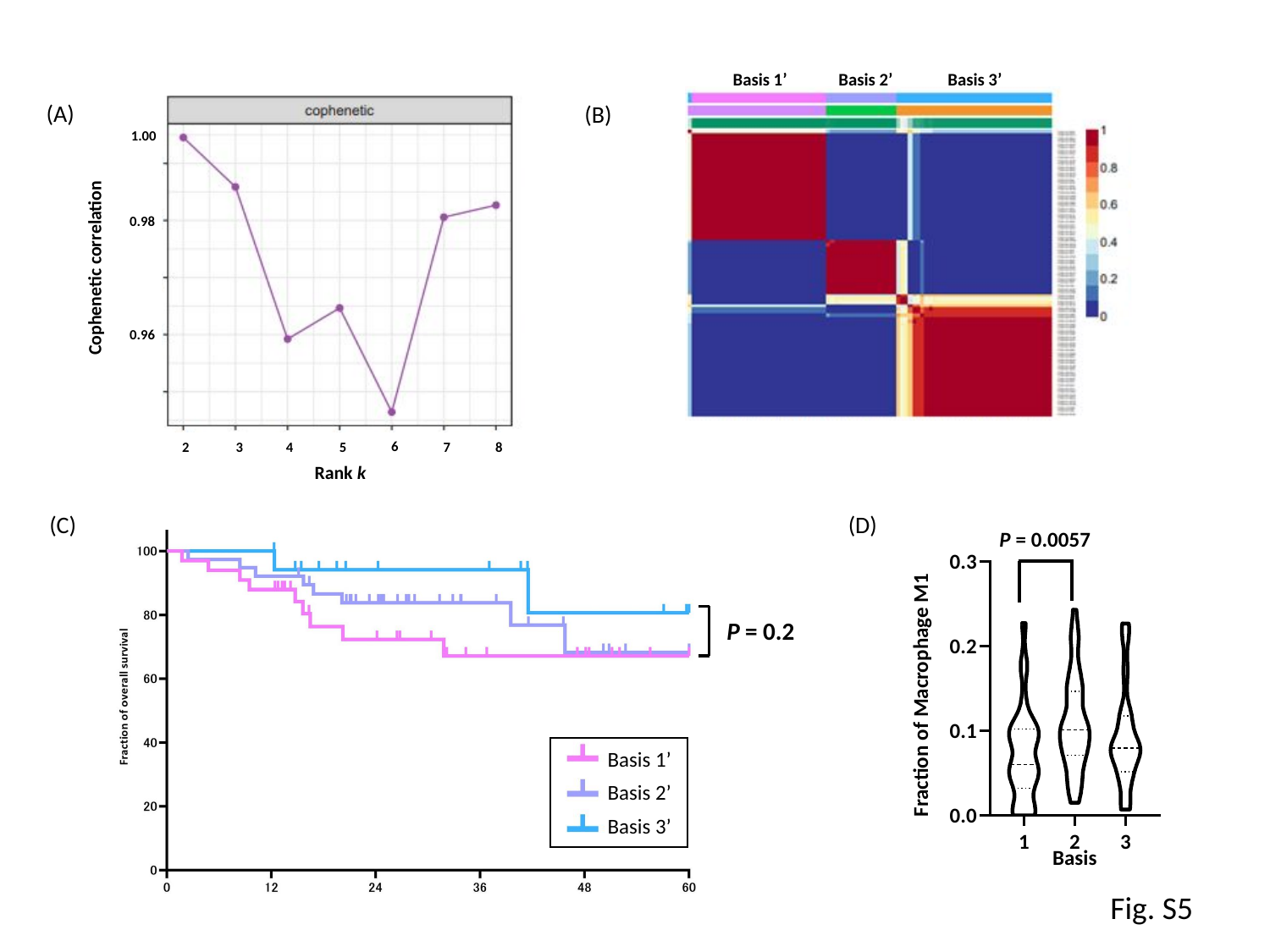

Basis 1’
Basis 3’
Basis 2’
(A)
(B)
1.00
0.98
Cophenetic correlation
0.96
6
2
3
4
5
7
8
Rank k
(C)
(D)
P = 0.0057
P = 0.2
Fraction of Macrophage M1
Basis 1’
Basis 2’
Basis 3’
Basis
Fig. S5

## Slide 7
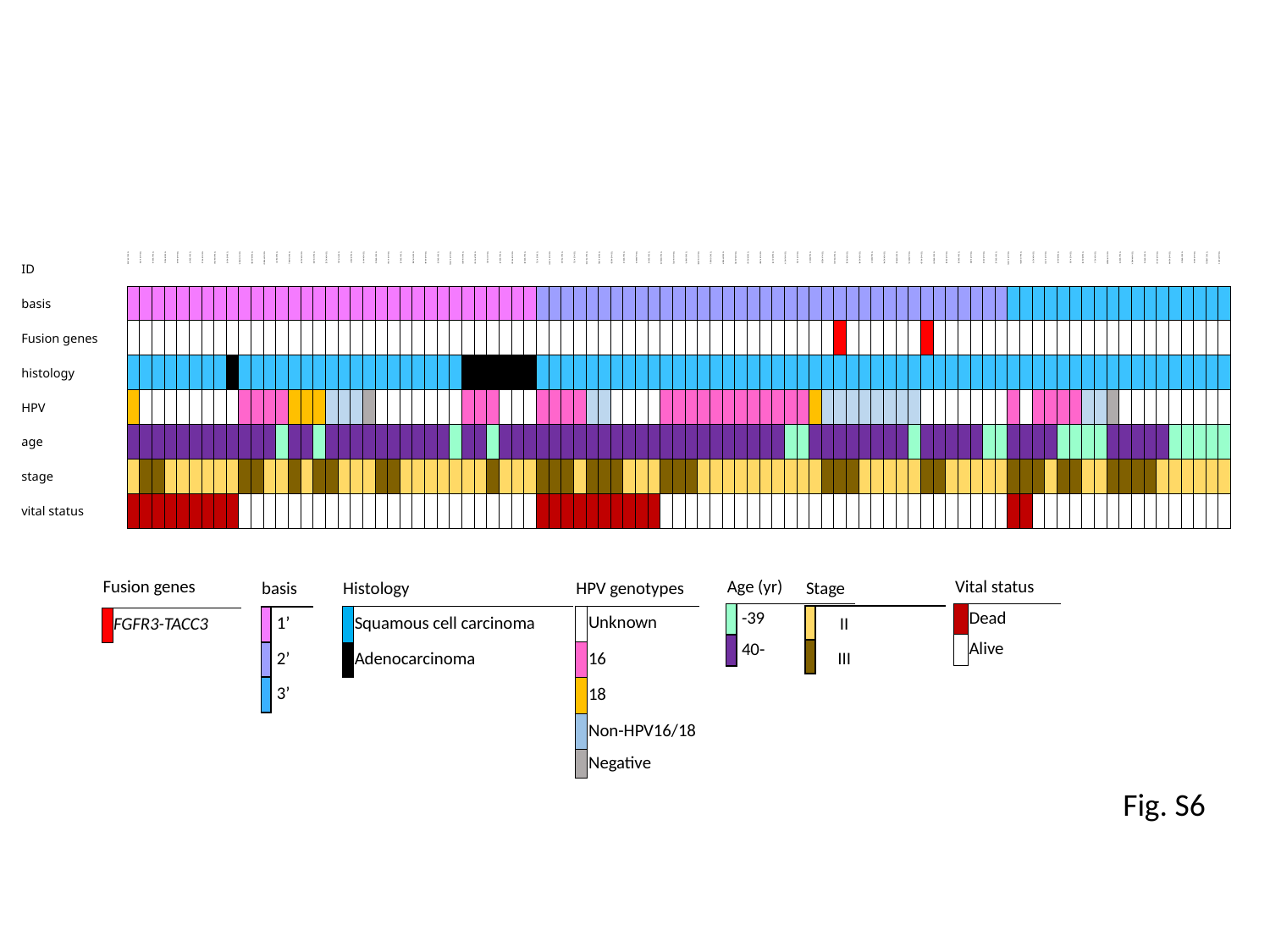

| ID | TCGA-C5-A1M6 | TCGA-C5-A7X3 | TCGA-VS-A9UL | TCGA-VS-A8EJ | TCGA-VS-AA62 | TCGA-VS-A94Y | TCGA-VS-A9UJ | TCGA-DS-A7WI | TCGA-VS-A9V5 | TCGA-DG-A2KK | TCGA-EA-A3QD | TCGA-MY-A5BD | TCGA-RA-A741 | TCGA-DG-A2KJ | TCGA-EA-A439 | TCGA-FU-A23K | TCGA-EK-A2GZ | TCGA-FU-A23L | TCGA-IR-A3LH | TCGA-EA-A410 | TCGA-VS-A8EC | TCGA-C5-A7UH | TCGA-VS-A9U5 | TCGA-FU-A3HZ | TCGA-VS-A9UB | TCGA-VS-A959 | TCGA-C5-A1MQ | TCGA-FU-A3EO | TCGA-LP-A7HU | TCGA-FU-A40J | TCGA-VS-A9UP | TCGA-VS-A9UO | TCGA-VS-A9UR | TCGA-C5-A7CL | TCGA-C5-A1MH | TCGA-C5-A2LZ | TCGA-C5-A7CJ | TCGA-C5-A1MK | TCGA-C5-A1BQ | TCGA-VS-A8EB | TCGA-VS-A9UI | TCGA-JW-A852 | TCGA-VS-A94X | TCGA-HM-A4S6 | TCGA-FU-A3TQ | TCGA-BI-A0VR | TCGA-FU-A3WB | TCGA-DG-A2KL | TCGA-MY-A5BF | TCGA-EA-A3HQ | TCGA-EA-A3HU | TCGA-C5-A3HD | TCGA-EX-A1H5 | TCGA-MU-A51Y | TCGA-C5-A1BJ | TCGA-JW-A5VJ | TCGA-JX-A3Q0 | TCGA-DS-A3LQ | TCGA-EA-A6QX | TCGA-EA-A3HR | TCGA-JW-A5VI | TCGA-EK-A2RA | TCGA-HM-A3JK | TCGA-JW-A5VG | TCGA-VS-A9UD | TCGA-VS-A8EH | TCGA-VS-A8QF | TCGA-VS-A958 | TCGA-C5-A8ZZ | TCGA-VS-A94Z | TCGA-VS-A9UC | TCGA-C5-A1MN | TCGA-UC-A7PG | TCGA-EK-A2PI | TCGA-C5-A7CH | TCGA-EA-A44S | TCGA-C5-A1BI | TCGA-EK-A2H0 | TCGA-EX-A3L1 | TCGA-DR-A0ZM | TCGA-VS-A950 | TCGA-MA-AA3X | TCGA-VS-A954 | TCGA-VS-A9UU | TCGA-VS-A94W | TCGA-VS-A8EI | TCGA-VS-A8EL | TCGA-ZJ-A8QQ | TCGA-MY-A913 |
| --- | --- | --- | --- | --- | --- | --- | --- | --- | --- | --- | --- | --- | --- | --- | --- | --- | --- | --- | --- | --- | --- | --- | --- | --- | --- | --- | --- | --- | --- | --- | --- | --- | --- | --- | --- | --- | --- | --- | --- | --- | --- | --- | --- | --- | --- | --- | --- | --- | --- | --- | --- | --- | --- | --- | --- | --- | --- | --- | --- | --- | --- | --- | --- | --- | --- | --- | --- | --- | --- | --- | --- | --- | --- | --- | --- | --- | --- | --- | --- | --- | --- | --- | --- | --- | --- | --- | --- | --- | --- |
| basis | | | | | | | | | | | | | | | | | | | | | | | | | | | | | | | | | | | | | | | | | | | | | | | | | | | | | | | | | | | | | | | | | | | | | | | | | | | | | | | | | | | | | | | | | |
| Fusion genes | | | | | | | | | | | | | | | | | | | | | | | | | | | | | | | | | | | | | | | | | | | | | | | | | | | | | | | | | | | | | | | | | | | | | | | | | | | | | | | | | | | | | | | | | |
| histology | | | | | | | | | | | | | | | | | | | | | | | | | | | | | | | | | | | | | | | | | | | | | | | | | | | | | | | | | | | | | | | | | | | | | | | | | | | | | | | | | | | | | | | | | |
| HPV | | | | | | | | | | | | | | | | | | | | | | | | | | | | | | | | | | | | | | | | | | | | | | | | | | | | | | | | | | | | | | | | | | | | | | | | | | | | | | | | | | | | | | | | | |
| age | | | | | | | | | | | | | | | | | | | | | | | | | | | | | | | | | | | | | | | | | | | | | | | | | | | | | | | | | | | | | | | | | | | | | | | | | | | | | | | | | | | | | | | | | |
| stage | | | | | | | | | | | | | | | | | | | | | | | | | | | | | | | | | | | | | | | | | | | | | | | | | | | | | | | | | | | | | | | | | | | | | | | | | | | | | | | | | | | | | | | | | |
| vital status | | | | | | | | | | | | | | | | | | | | | | | | | | | | | | | | | | | | | | | | | | | | | | | | | | | | | | | | | | | | | | | | | | | | | | | | | | | | | | | | | | | | | | | | | |
| Fusion genes | | | |
| --- | --- | --- | --- |
| | FGFR3-TACC3 | | |
| basis | | |
| --- | --- | --- |
| | 1’ | |
| | 2’ | |
| | 3’ | |
| Histology | | | | | | | | | | |
| --- | --- | --- | --- | --- | --- | --- | --- | --- | --- | --- |
| | Squamous cell carcinoma | | | | | | | | | |
| | Adenocarcinoma | | | | | | | | | |
| Vital status | | | |
| --- | --- | --- | --- |
| | Dead | | |
| | Alive | | |
| HPV genotypes | | | | | |
| --- | --- | --- | --- | --- | --- |
| | Unknown | | | | |
| | 16 | | | | |
| | 18 | | | | |
| | Non-HPV16/18 | | | | |
| | Negative | | | | |
| Age (yr) | | | | | | |
| --- | --- | --- | --- | --- | --- | --- |
| | -39 | | | | | |
| | 40- | | | | | |
| Stage | | |
| --- | --- | --- |
| | II | |
| | III | |
Fig. S6
