# Supplementary material for: Rare FGFR fusion genes in cervical cancer and transcriptome‐based subgrouping of patients with a poor prognosis
Source: Cancer Med. 2023 Aug 3;12(17):17835–48. doi: 10.1002/cam4.6415 (PMC10524028; doi:10.1002/cam4.6415)
Supplement: Supplementary file 3 — Data S1. Supporting material and methods [file CAM4-12-17835-s003.docx]

**Supporting material and methods**

**Rare FGFR fusion genes in cervical cancer and transcriptome-based subgrouping of patients with a poor prognosis**

Hiranuma K et al.

**Supplementary methods:**

**Patient rights and informed consent**

All patients provided written informed consent for the collection of samples and subsequent analyses. The Institutional Review Board of the National Cancer Center Research Institute approved this study (2017-136). For patients diagnosed between 2002 and 2020, informed consent for the use of samples in research was obtained during their first visit to our hospital. Information obtained using the samples collected is listed on the hospital’s website (https://www.ncc.go.jp/jp/biobank/). Patients were free to revoke their consent at any time. Only samples from patients who did not revoke their consent were included.

**HPV genotyping by Sanger sequencing**

HPV genotyping was performed for the 116 samples. Genomic DNA (10 ng) was amplified via PCR using TaKaRa Taq DNA polymerase (Takara Bio Inc., Shiga, Japan) for two distinct HPV genomic regions. The HPV E6/E7 homologous region was amplified using the pU-1M/pU2R primer set, and the region containing the HPV L1 gene was amplified using the GP5+/GP6+ and the L1C1/L1C2M primer sets (Supplementary Table 2). PCR reactions were performed using the TaKaRa PCR Human Papillomavirus Typing Set (Takara Bio Inc.). PCR products were purified using the NucleoSpin Gel (Takara Bio Inc.) or PCR Clean-up kit (Takara Bio Inc.). Sanger sequencing was performed using an ABI 3130xl DNA Sequencer (Applied Biosystems), according to the manufacturer’s instructions. The similarity between the obtained sequences and various HPV genotypes in the GenBank database was determined using Basic Local Alignment Search Tool (BLAST) (https://blast.ncbi.nlm.nih.gov/Blast.cgi).

**Detection of high-risk HPV types and identification of the HPV back splice junction in cervical cancer tissues**

To assess the frequency of HPV-positive cases, we performed in situ hybridization for HPV detection (HPV-ISH) using HPV-III High-Risk probes (Roche Diagnostics, Mannheim, Germany) according to the manufacturer’s instructions. This assay can detect high-risk HPV genotypes, including HPV-16, 18, 31, 33, 35, 45, 52, 56, 58, and 66, in cervical cancer specimens ^1^.

To screen for the presence and integration of circular RNA (circRNA) from HPV, we used the vircircRNA pipeline in HPV 16-positive and HPV 18-positive cases from RNA-Seq datasets (<https://github.com/jiwoongbio/vircircRNA>) ^2^. HPV 16 and HPV 18 gff reference files were downloaded from National Center for Biotechnology Information (https://ftp.ncbi.nlm.nih.gov/genomes/Viruses/).

The overexpression of HPV E6 and E7 oncoproteins is critical and necessary for HPV-mediated cervical carcinogenesis, and binding of E6 oncoproteins accelerates the ubiquitin-mediated degradation of the TP53 tumor suppressor protein and other targets ^35,36^. We used the vircircRNA pipeline (<https://github.com/jiwoongbio/vircircRNA>) ^37^ to determine the expression of the HPV E6 and E7 regions and integration sites. Next, we examined whether the expression of HPV E6/E7 is also associated with the prognosis of patients with cervical cancer. In both the present cohort and TCGA dataset, HPV E6/E7-positive cases tended to have a better prognosis than HPV E6/E7-negative cases, although the difference was not significant (*P* = 0.09 and *P* = 0.19, respectively; Supplementary Figure 1).

Supplementary Figure 1. Kaplan–Meier survival curves according to HPV E6/E7 status. (A) Recurrence-free survival in HPV E6/E7-positive (blue) and -negative (gray) cases in the present cohort. Of the 65 cases that underwent adjuvant therapy, 52 with HPV 16/18-positive status were analyzed. (B) Overall survival of HPV E6/E7-positive (blue) and -negative (gray) cases in TCGA dataset. Of the 89 cases, 22 with HPV 16/18-positive status were analyzed. Cases surviving <1 year and those with stage I/IV were excluded.

**Supplementary figure legends:**

**Figure S1.** Identification of the *FGFR3-TACC3* fusion. (A) Detection of *FGFR3-TACC3* fusions by PCR. (B-F) Electropherogram for Sanger sequencing of genomic fragments encompassing the *FGFR1-ADAM9*, *FGFR3-TACC3*, and *GOPC-ROS1* breakpoint junctions.

**Figure S2.** Frequency of patients with *FGFR1/2* fusion genes in various cancer types from two public datasets (cBioPortal and C-CAT). (A) *FGFR1* fusion cases. (B) *FGFR2* fusion cases.

Breast cancer: BRC; head and neck cancer: HNC; central nervous system: CNS; brain tumor: BRT; prostate cancer: PRC; colorectal cancer: CRC; ovarian cancer: OVC; lung cancer: LUC; pancreatic cancer: PAC; hepatobiliary cancer: HBC; hepatocellular cancer: HCC; kidney cancer: KDC; cervical cancer: CEC; thyroid carcinoma: THC; esophagogastric cancer: ESC; soft tissue tumor: SOT; uterine carcinoma: UTC; bladder cancer: BLC; urinary carcinoma: URCA.

**Figure S3.** mRNA clustering analysis of the present cohort. Gene expression values were obtained from RNA-seq data of 32 characteristic genes shown in Table S6 from Ref #7 in 108 cervical cancer cases (except neuroendocrine cancer). Three clusters (keratin-high, keratin-low, and adenocarcinoma) are shown.

**Figure S4.** Four divided classes and gene aberration profiles of 65 cervical cancer cases. Bases 3/4 are highly relapsed clusters with non-SCC histology, whereas Bases 1/2 are less frequently relapsed clusters with SCC histology.

**Figure S5.** NMF clustering analysis of 89 TCGA cervical cancer cases performed using 91 differentially expressed genes (DEGs). (A) Cophenetic correlation coefficients for the hierarchically clustered matrices. High cophenetic correlations were observed for *k* = 2 and *k* = 3 classes. (B) Consensus matrix for *k* = 3 is shown. (C) Kaplan–Meier survival curves of the 89 patients with cervical cancer. (D) Fraction of M1 macrophages in the three Bases.

**Figure S6.** Clinicopathological characteristics of each Basis group of 89 TCGA cervical cancer cases.

**References**

1 Hirose S, Murakami N, Takahashi K, et al. Genomic alterations in STK11 can predict clinical outcomes in cervical cancer patients. *Gynecol Oncol*. 2020;156:203-210.

2 Zhao J, Lee EE, Kim J, et al. Transforming activity of an oncoprotein-encoding circular RNA from human papillomavirus. *Nat Commun*. 2019;10:2300.
